# Patient Experience of Integrated Care: Findings from a Cross-Sectional Study Involving People with Rheumatic and Musculoskeletal Diseases

**DOI:** 10.5334/ijic.6616

**Published:** 2023-05-10

**Authors:** Nicola Spezia, Serena Barello, Chiara Torri, Antonella Celano, Guendalina Graffigna

**Affiliations:** 1 EngageMinds HUB –Consumer, Food & Health Engagement Research Center, Department of Psychology, UniversitàCattolica del Sacro Cuore, Milan, Italy; 2 Apmarr APS (Italian Association of People with Rheumatological and Rare Diseases), Lecce, Italy

**Keywords:** Integrated care, patient experience, rheumatic and musculoskeletal diseases (RMDs), Italy, person-centred care, health service delivery, patient engagement, value-based healthcare

## Abstract

**Introduction:**

Although the patient experience of integrated care has been documented for several chronic conditions, little is known in the context of rheumatic and musculoskeletal diseases (RMDs). This study provides a first overview of the patient experience of integrated care according to the perspective of people living with RMDs in Italy.

**Methods:**

A cross-sectional survey was administered to 433 participants who reported their experiences together with the importance assigned to different attributes of integrated care. Explorative factor analysis (EFA) and non-parametric ANOVA and ANCOVA statistical tests were employed to account for the differences in the answers provided by sample subgroups.

**Results:**

Two factors (namely, “Person-centred care” and “Health service delivery”) were extracted in the EFA. Participants attributed high importance to both of them. Overall positive experiences were reported only for Person-centred care. The delivery of health services instead received a poor evaluation. Significantly worse experiences were observed for women and people that were either older, unemployed, with comorbidities or lower self-reported health, or less engaged in their healthcare management.

**Conclusions:**

Italians with RMDs described integrated care as an important approach to care. However, further effort is needed to allow them to perceive an actual benefit from integrated care practices. Specific attention should be paid to disadvantaged and/or frail population groups.

## Introduction

Rheumatic and musculoskeletal diseases (RMDs) rank as one of the most prevalent chronic conditions affecting approximately 1,71 billion people worldwide [1]. RMDs are autoimmune and inflammatory chronic diseases that affect joint tendons, ligaments, bones, and muscles. More than 200 distinct RMDs exist, among them the most common are different types of arthritis [2]. Common symptoms include pain, stiffness, and swelling in the joints, but RMDs can also affect other areas of the body, such as internal organs. In the most severe conditions, some routine activities such as bathing, dressing, or walking can cause pain and be difficult or even impossible. Treatment plans for people living with RMDs usually include medications, regular exercise, a healthy diet, stress management, and rest. Besides, these people are taken care of by different primary and secondary care health professionals such as general practitioners (GPs), rheumatologists, physiotherapists, orthopaedists, and occupational therapists. However, even with appropriate treatment plans, a significant proportion of people with RMDs are likely to deal with continuous daily symptoms during their lives with negative impacts on their physical, mental, and social health-related quality of life [3].

To face the challenges posed by the growing incidence among the population of chronic conditions such as RMDs, the World Health Organization (WHO) advocates an integrated approach to healthcare [4]. Based on multidisciplinary working and the continuity and coordination of different levels of care [5], integrated care aims to go beyond the fragmentation and isolation of health services. In an integrated care setting, these services are designed and delivered in response to chronic and continuous patient health needs besides episodic ones [6]. Integrated care interventions have been proven to enhance the clinical outcomes and quality of life of chronic patients [7,8], as well as reduce their hospital admission rates and length of stay [9]. Furthermore, growing evidence shows that integrated models of health service delivery might foster patients’ access to care and improve their perceived quality of care and satisfaction [10]. In this view, integrated care represents a key enabler of person-centred care, allowing patients to achieve the outcomes that are most important for them [5].

While the introduction and implementation of integrated care service models have been systematically documented for a number of chronic conditions such as chronic obstructive pulmonary disease, heart failure, chronic kidney disease, diabetes, and mental illnesses [11,12,13], little is known in the context of RMDs. Particularly, limited evidence about the patient experience of integrated care reported by people with RMDs exists. Patient experience describes patients’ perceptions of the various interactions they have with the health system. In this way, patient experience provides an assessment of the quality of different elements of health delivery directly from the users’ viewpoint. For this reason, patient experience has been indicated as a crucial measure of integration and more broadly of healthcare quality [14,15]. This measure can be further enhanced by considering patients’ priorities and segmenting the patient population. Patients’ priorities can be assessed by observing which experiences they value as more important and therefore defining “what matters most to them” [16]. Patient segmentation instead allows the identification of different subgroups, which may account for specific experience profiles and priorities [17,18,19]. Segmentation typically considers different clinical and socio-demographic variables, however, there is an emerging need to take into account also the psychological factors that may affect people’s needs and expectations towards the received care. Between them, patient engagement can be described as a continuum of profiles describing different possible ways to interact with the healthcare system [20]. The profile of patient engagement is particularly relevant as it may mediate the measurement of patient experience representing therefore a key variable to characterise a population [20,21,22].

As mentioned before, little research has focused on the patient experience of integrated care in the context of RMDs. Most of this evidence consists of studies describing the experience of people living with a specific RMD and in a restricted setting (such as a singular hospital or project) [e.g. 23,24,25]. However, to the best of our knowledge, less attention has been given to more comprehensive and stratified analysis.

In Italy, the challenges of chronic disease prevention and management have been addressed on a national level by the National Chronicity Plan, which was launched in 2016. This policy provides a set of general strategies and guidelines aiming at improving the health protection and quality of life of chronically ill people [26]. These include the recommendation to implement an integrated approach in organising and delivering health services for chronic conditions, including RMDs. However, no specific strategies or programmes are indicated in the National Chronicity Plan. This is due to the fact that, while the broad goals and directives are set at the national level, most of the resources and the strategic and operational plans are managed on a regional basis. Italy is divided into 20 administrative regions, each of which has its regional health service interpreting and applying the national directives independently, with high degrees of freedom. This also resulted in significant historical differences in the provision and quality of care across the country, especially when comparing the regions of the north, centre, and south [27,28]. Since also the implementation of integrated care practices is left to independent regional or local initiatives, a national picture of integrated care for Italians with RMDs is needed.

Considering these premises, this study provides a first overview of the patient experience of integrated care in the context of RMDs in Italy. In doing so, the objective was also to identify the priorities concerning integrated care for people with RMDs, as well as compare the experience profiles and priorities of different subgroups.

## Research Methods

### Setting and population

This study was funded by the national-based Italian patient organisation “Apmarr APS” (Association of People with Rheumatological and Rare Diseases).

An online cross-sectional self-administered survey was distributed from June to September 2021 to a sample of Italian people with RMDs. Part of the participants were recruited by a professional panel provider (Toluna Inc., https://tolunacorporate.com), the remaining by Apmarr APS. This latter shared — through its mailing list and social media — the invitation to take part in the study and to complete the online survey uploaded on the platform Qualtrics®. Inclusion criteria were: age 18 years and older and diagnosed with one or more RMDs. The study protocol was approved by the Ethics Committee of the authors’ university. All people involved in the study gave informed consent to participate.

### Measures

The study questionnaire (Appendix) consisted of three main sections:

1. **Patient experience of integrated care:** 19 items developed ad hoc by the authors based on the literature and advice provided by Apmarr APS. The first six items focus on the person-centred care approach, which should be central in any integrated care setting [4,29]. Specifically, we considered the following key principles of person-centred care: holistic and equitable care, involvement in healthcare decision-making, continuity of care, and support from health professionals [30].
  The remaining items instead relate to a selection of the features of health service delivery that are enablers of effective integrated care implementation [31]. In particular, we focused on those features within the scope of primary and secondary care that we believed to be the most easily appreciable from a patient perspective. In this sense, we investigated the proactivity, accessibility, responsiveness, flexibility, and degree of digitalisation of healthcare services. For service proactivity, the capacity of health professionals to reach out to patients to invite them for a medical check-up or follow-up examination was considered. Accessibility was analysed in terms of the closeness between rheumatology clinics and patients’ homes and the possibility to be visited at home by health professionals. Responsiveness considered the waiting times for medical examinations. Flexibility contemplated the possibility to choose a specific doctor and the preferred (according to the patient’s schedule) day and time for a rheumatology examination. Finally, for the degree of digitalisation of services, the possibility to book a rheumatology visit online, effectively use an electronic health record, and perform a medical examination via video call were studied.
  Every item in this section included two questions. The first question investigated the survey respondents’ experiences. The second one, to determine patients’ priorities, analysed the importance they attributed to the item. Both questions’ response options consisted of a five-point Likert scale (from “never” to “always” for experience and from “not important” to “very important” for importance).
2. **Patient Health Engagement scale (PHE-s®):** A validated scale that comprises five items to measure respondents’ actual engagement in their healthcare management [32]. The PHE-s® is based on the PHE model that was developed by embracing the notions and paradigms of health psychology. It is an evidence-based psychological theory developed through a systematic and in-depth study of people’s illness experiences by means of narrative qualitative research. According to the PHE model, becoming engaged in healthcare management means being more and more resilient towards the personal health condition and the care requirements to effectively manage it. The PHE-s ® allows to identify people’s health engagement current status, clustering respondents into four main patient engagement profiles along a psychological continuum from low to high patient health engagement [20]. The first profile of patient engagement (namely, “Blackout” that is a label directly coming from the patients’ narratives), mainly occurs when patients feel vulnerable because of a critical event, such as a diagnosis, new symptomatology, a disease relapse, and the need to assume new lifestyles to manage their illness condition. In this phase, patients seem psychologically frozen and feel totally paralysed.
  Patients in the Arousal profile – which is the next one in the patient engagement continuum – have acquired an initial awareness of their health condition, but still have superficial knowledge about how to manage it effectively. They cannot adapt to it and consider their new health status as part of their daily lives. These patients often report that they are hypervigilant over their bodies signals.
  When people succeed in the process of emotional regulation and coping with the illness condition, they experience the Adhesion profile. In the latter, patients have developed a good acceptance of their disease and have overcome the major psychological stress connected to its onset. Moreover, they report being aware of their health status and its impact on their lives and life habits. They are also increasingly knowledgeable about how to effectively manage the disease and treatments.
  People experiencing the Eudaimonic Project profile – the last one described by the PHE Model – have elaborated and accepted their own “patient identity” as one of the many shades in a person’s life. This means that they have become completely aware of their disease and its implications in terms of changed life habits and therapeutic requirements. These patients can integrate – in a more synergic manner – their health and disease management into their life goals. In this phase, people become active agents in seeking a satisfying quality of life, even if living with a disease. This perspective makes them able to embrace a more positive approach to life.
  The PHE-s® is measured on a seven-point scale, a specific algorithm provides the final score indicating in which of the four engagement positions the respondent is located [32]. Identifying the patient engagement profile is crucial to orient healthcare interventions in a way they are more personalised according to the patients’ expected role in their care journey and their actual psychosocial and support needs. However, it is necessary to highlight that the patient’s choice to actively participate (or not) in his/her own healthcare management is also situational (i.e. not depending solely on the engagement profile). In this view, healthcare systems must always be ready to sustain patients in whatever choice they make about the role they want to play in their care journey.
3. **Respondent characteristics:** 10 items collecting the following descriptive variables: sex, age, region of residence, marital status, education, employment, distance between home and clinics, years from diagnosis, presence of other diseases (not RMDs), and self-reported health (evaluated using a visual analogue scale with a range of scores from 0 to 100).

To provide content and face validation, Apmarr APS established a multidisciplinary board composed of rheumatology practitioners and expert patients that reviewed the questionnaire during an online meeting in May 2021. Specifically, board members were asked to evaluate the content, clarity, and readability of each survey item expressing “out loud” their thoughts and establishing an open discussion. While no specific concern about survey content was raised by the board, the wording of some items was revised to improve their readability.

### Data Analysis

The data analysis consisted of four steps. In step one, we reported the descriptive characteristics of the sample using frequency analysis.

In step two, an explorative factor analysis (EFA) was performed to investigate whether significant multi-item scales (i.e. factors) could be defined. The factors account for the correlation among different survey questions. In this way, they should reflect the underlying theoretical structure of the questionnaire, thus providing further validation to the assumptions behind its development [33]. Therefore, in our study, factors were interpreted as the dimensions of the patient experience of integrated care investigated by the survey. The EFA included all 19 items in the first section of the survey. For each item, only the experience question was considered. The EFA was configured specifically to analyse ordinal data [34,35]: polychoric correlation to estimate the questions’ correlations, minimum rank factor analysis as the factor extraction method, parallel analysis to determine the number of factors, and Promin as factor rotation method. Factor loadings ≥ .4 were considered significant. The produced factors were subjected to reliability analysis estimating the ordinal alpha coefficient [36].

In step three, a descriptive analysis of the experience and importance attributed to integrated care by the sample was performed. To do so, we developed a simple but functional framework combining the measure of experience and importance. This framework consists of a matrix where patient experience is reported on the x-axis and level of importance is posed on the y-axis. In this way, four quadrants are created: on the right end (positive experience), the primary (high importance) and secondary (low importance) strengths of the delivered integrated care are highlighted. On the left end (negative experience), a hierarchy of the priorities of intervention for quality improvement is established (moving from high to low importance). We implemented our framework using the survey items and the factors extracted in the EFA.

Finally, in step four, the differences in the reported experience and importance attributed to integrated care among several subgroups of the sample were assessed. For this purpose, we implemented Kruskal-Wallis H (or non-parametric ANOVA) and Quade’s (or non-parametric ANCOVA) statistical tests. Specifically, this latter was used when it was necessary to statistically control for some confounding variables. The choice of non-parametric tests was due to the ordinal nature of the data [37]. Furthermore, Scheffe’s adjustments were made when comparing multiple (more than two) subgroups at the same time. This post-hoc test is especially recommended when the subgroups have different sizes [38]. Subgroups have been defined using the different ordinal levels of the collected descriptive variables. We assessed the differences among the sample subgroups considering the factors extracted in the EFA. Furthermore, we also performed a qualitative (i.e. without using statistical tests) comparison of the four subgroups of the sample with different engagement profiles identified through the PHE-s®.

To perform the statistical analyses, we coded the responses to the items about integrated care with a score ranging from 1 to 5. Mean scores were also computed considering the items belonging to the same factor extracted in the EFA. Statistical analyses have been performed with the software IBM SPSS® version 27, except for the EFA that was run on the software Factor version 11. The statistical significance level was set at 5%.

## Results

### Sample description

The sample involved in this study consisted of 433 people diagnosed with one or more RMDs. The descriptive characteristics of the sample are shown in Table 1. Women represented 71% of the sample, whereas 57% of the respondents were 50 years old or older. Around 44% of the participants lived in northern Italy, 19% in the centre, and 37% in the south. Almost 88% of the respondents had a high school degree, 66% were employed (13% have retired from work), and 75% were married. More than 66% of the participants lived less than 20 km away from the clinics where they received treatments, while 11% lived more than 50 km far from them. 79% of the sample has been diagnosed with one or more RMD for at least three years, while almost 59% of the respondents suffered also from other types of diseases (not RMDs). The mean self-reported health was equal to 59 (on a scale from 0 to 100). About 42% of the participants reported a low health engagement profile (i.e. Blackout or Arousal).

**Table 1:** Sample characteristics (N = 433).


	N	%

**Sex**		

Female	307	71%

Male	126	29%

**Age**		

65+	53	12%

50–64	193	45%

35–49	133	31%

18–34	54	12%

**Region of residence**		

Northern Italy	190	44%

Central Italy	84	19%

South Italy	159	37%

**Marital status**		

Married	288	66%

Divorced	98	23%

Single	47	11%

**Education**		

University degree	143	33%

High school diploma	237	55%

Middle school diploma	53	12%

**Employment**		

Employed	287	66%

Retired	55	13%

Unemployed	91	21%

**Distance between home and clinics**		

More than 50 km	47	11%

Between 20 km and 50 km	98	23%

Less than 20 km	288	66%

**Years from diagnosis**		

More than 10	110	26%

Between 3 and 10	230	53%

Less than 3	93	21%

**Presence of other diseases**		

Yes	254	59%

No	179	41%

**Self-reported health** *(mean = 59 points, standard deviation = 22 points)*		

High (>75 points)	109	25%

Medium (≥50 and ≤75 points)	219	51%

Low (<50 points)	105	24%

**PHE-s® scores (engagement position)**		

Blackout	34	9%

Arousal	146	33%

Adhesion	220	51%

Eudaimonic Project	33	7%


### Explorative factor analysis

The EFA produced two factors explaining around 69% of the variance shared among the items included in the analysis. Every item loaded significantly to either one of them (Table 2). Particularly, since the first six items loaded significantly to the first factor, this was named “Person-centred care”. All the remaining items instead loaded significantly to the second factor and therefore this was named “Health service delivery”. Both factors showed very high internal consistency with Ordinal α respectively of .90 and .94.

**Table 2:** Explorative factor analysis: factor loadings.


SURVEY ITEMS	FACTOR ONE: PERSON-CENTRED CARE	FACTOR TWO: HEALTH SERVICE DELIVERY

Q1: holistic care	.833	.075

Q2: health services without discrimination or distinctions	.867	–.057

Q3: involvement in healthcare decision-making	.780	.091

Q4: health services with continuity of professionals	.897	–.031

Q5: support from GP	.813	–.035

Q6: support from rheumatology specialists	.781	.046

Q7: proactivity of GP	.035	.881

Q8: proactivity of rheumatology specialists	–.011	.897

Q9: closeness of rheumatology clinics	.074	.715

Q10: home visit by GP	.097	.666

Q11: home visit by rheumatology specialists	–.009	.736

Q12: waiting time for GP appointment	.103	.665

Q13: waiting time for rheumatology examination	–.081	.949

Q14: specific doctor for rheumatology examination	–.139	.984

Q15: preferred day and time for rheumatology examination	.078	.679

Q16: book rheumatology examination online	.170	.545

Q17: useful electronic health record	.065	.813

Q18: GP examination online	–.065	.861

Q19: rheumatology examination online	–.100	.983


*Note*: Q(n): Question (item) number.

### Experience and importance attributed to integrated care

**Figure 1:**
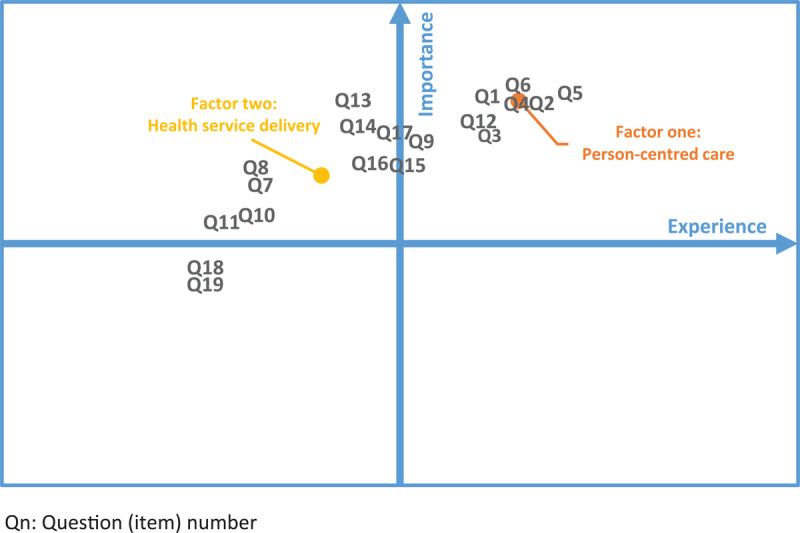
Experience-Importance matrix: entire sample.

The experience-importance matrix of the entire sample is plotted in Figure 1. Medium/high or high importance was assigned to almost all items, leaving the bottom quadrants of the matrix nearly empty. In this sense, only the two items investigating the possibility to perform medical examinations via video call were considered less important by the study participants. Considering the factors produced in the EFA, Person-centred care resulted to be the most important for the respondents. Furthermore, overall positive experiences were reported for this factor. Health service delivery instead received a poor evaluation, especially when considering the items about the accessibility, proactivity, and the degree of digitalisation of services.

### Differences across subgroups

The outputs of Kruskal-Wallis H and Quade’s tests performed to assess the differences in the reported experience and importance attributed to integrated care between the subgroups of the sample are presented in Table 3. No statistically significant differences were observed for the subgroups defined using the following variables: region of residence, marital status, education, distance between home and clinics, and years from diagnosis. The results of the two groups and multiple groups comparisons are reported in Table 4 showing exactly where significant differences occurred.

**Table 3:** Differences across subgroups: Kruskal-Wallis H and Quade’s tests.


VARIABLE	FACTOR ONE: PERSON-CENTRED CARE		FACTOR TWO: HEALTH SERVICE DELIVERY		CONTROLLED VARIABLES (CONFOUNDERS)
	
	EXPERIENCE	IMPORTANCE	EXPERIENCE	IMPORTANCE	

**Sex**	–	H(1) = 45.292 p < .001	H(1) = 20.201 p < .001	H(1) = 6.998 p = .008	None

**Age**	–	–	H(3) = 24.216 p < .001	–	None

**Region of residence**	–	–	–	–	None

**Marital status**	–	–	–	–	Age

**Education**	–	–	–	–	Sex, age, region of residence

**Employment**	–	–	F(2,430) = 4.266 p = .015	–	Sex, age, region of residence, education

**Distance between home and clinics**	–	–	–	–	None

**Years from diagnosis**	–	–	–	–	Age

**Presence of other diseases**	–	F(1,431) = 4.944 p = .027	F(1,431) = 4.187 p = .041	–	Age

**Self-reported health**	F(2,430) = 5.267 p = .005	–	F(2,430) = 7.488 p < .001	F(2,430) = 3.254 p = .040	Sex, age, region, education, employment, years from diagnosis, presence of other diseases


*Note*: H: Kruskal-Wallis H test, F: Quade’s test, –: not significant (p >.05).

**Table 4:** Differences across subgroups: Two groups and multiple groups comparisons.


VARIABLE	FACTOR ONE: PERSON-CENTRED CARE		FACTOR TWO: HEALTH SERVICE DELIVERY	
	
	EXPERIENCE	IMPORTANCE	EXPERIENCE	IMPORTANCE

**Sex**				

*Female vs. Male*	–	Δ = .50 p < .001	Δ = –.48 p < .001	Δ = .18 p = .008

**Age**				

*65+ vs. 50–64*	–	–	–	–

*65+ vs. 35–49*	–	–	–	–

*65+ vs. 18–34*	–	–	Δ = –.63 p = .009	–

*50-64 vs. 35–49*	–	–	Δ = –.43 p = .004	–

*50-64 vs. 18–34*	–	–	Δ = –.66 p < .000	–

*35-49 vs. 18–34*	–	–	–	–

**Employment**				

*Unemployed vs. Employed*	–	–	Δ = –.52 p = .042	–

*Unemployed vs Retired*	–	–	–	–

*Employed vs Retired*	–	–	–	–

**Presence of other diseases**				

*Yes vs. No*	–	Δ = .14 p = .027	Δ = –.22 p = .041	–

**Self-reported health**				

*Low vs. Medium*	–	–	Δ = –.40 p = .035	–

*Low vs. High*	Δ = –.42 p = .006	–	Δ = –.58 p = .001	–

*Medium vs High*	–	–	–	–


*Note*: Δ: difference in the mean scores between the two subgroups, –: not significant (p >.05).

Women evaluated both factors (i.e. Person-centred care and Health service delivery) as more important compared with men. However, their experience of Health service delivery was significantly worse. The experience of Health service delivery was also markedly worse for older participants (aged 50 and older) compared with younger ones. This was also true for unemployed respondents compared to employed ones. People who suffer from other types of diseases besides RMD one(s) attributed more importance to Person-centred care. However, their experience of Health service delivery was slightly worse. Finally, people whit lower self-reported health described poorer experiences concerning both Person-centred care and Health service delivery compared with those with high self-reported health.

**Figure 2:**
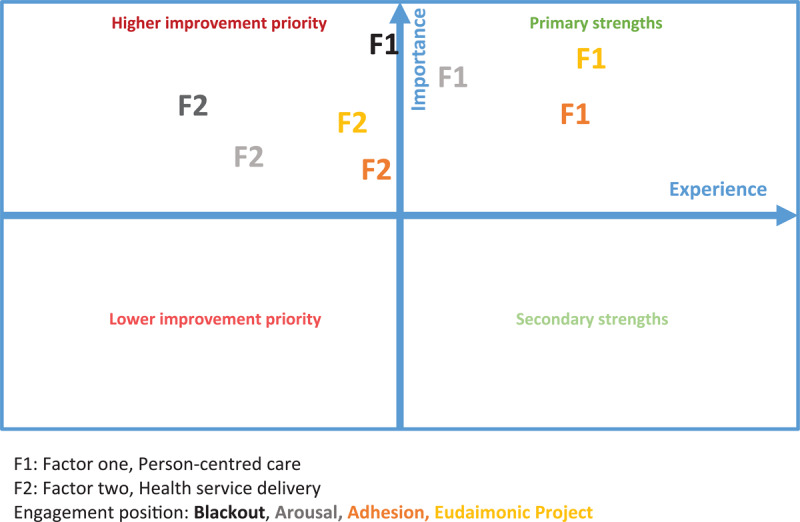
Experience-Importance matrix: subgroups with different health engagement profiles.

Participants with the lowest profile of health engagement (i.e. Blackout) attributed the highest importance to both Person-centred care and Health service delivery. However, they described the poorest experiences for both factors. Importance decreases and experiences improve for both factors moving throughout the next two engagement positions (i.e. Arousal and Adhesion). Similar to less engaged ones, instead, participants with the highest engagement profile (i.e. Eudaimonic Project) attributed higher importance to both factors. Finally, these respondents reported the best experiences for Person-centred care but not for Health service delivery. Figure 2 shows the experience-importance matrix of the four subgroups with different health engagement profiles.

## Discussion

This study aimed to provide a first overview of the patient experience of integrated care in the context of RMDs. We studied patient experience considering also what people evaluated as the most important. We combined the experience and importance measures to generate more accurate and actionable information, allowing the identification of the priorities of intervention for quality improvement. We segmented the patient population by comparing the responses provided by different subgroups defined according to the descriptive variables of the sample. Besides, we also considered the respondents’ profiles of health engagement in their care management, as this represents a central factor defining the behavioural attitudes and needs of patients [20]. We performed segmentation to identify the specific needs and concerns of different subgroups, providing additional information to design targeted initiatives. In our study, we focused on the key dimensions of person-centred care and on a selection of the features of health service delivery enabling effective integrated care implementation. This research was conducted in Italy. We verified whether the regional differences in the organisation, delivery, and quality of Italian healthcare reflected also on the patient experience of integrated care of people living with RMDs.

Almost all the investigated attributes of integrated care were described as important or very important, confirming the relevance of this approach also for Italians with RMDs. Participants described receiving person-centred care, which was also the most important thing for them. Lower overall importance was attributed to the delivery of health services. However, poor experiences were described for all the features of service delivery included in the analysis. Therefore, our findings suggest that, as far as improvement actions can be undertaken in the individual areas following a priority logic, only comprehensive interventions to enhance service delivery will allow Italians with RMDs to benefit from effective integrated care.

Only the possibility to have a medical examination via video call with the GP or a rheumatology specialist was considered not so important by study participants. Little evidence from Italy was found to better contextualise this result. In one recent study [39], teleconsultations were largely used and preferred to traditional face-to-face assessments by almost 2/3 of the patient sample. However, this research involved only a single hospital (a third-level rheumatology centre in Northern Italy) making it difficult to compare its results with those from a national sample. Moreover, it was carried out during the early stages of the COVID-19 outbreak when a strict lockdown was imposed on the whole country. In this period, most of the in-person consultations stopped and it is also plausible that patients were more concerned about the new and relatively unknown disease opting for teleconsultations over traditional ones. It is less clear what is the role and importance of telemedicine in the context of RMDs during the current post-pandemic phase, as shown by some contrasting findings reported in the international literature [40,41,42,43]. For these reasons, additional and continuous research on the implementation and relevance for people with RMDs of teleconsultation and other telemedicine applications should be carried out.

Segmenting the sample, we identified some subgroups who reported worse experiences of integrated care, especially when evaluating service delivery. These included people in a condition of frailty (either older, with comorbidities, or with lower self-reported health), which should be considered with special attention when designing and implementing integrated care initiatives and programmes [12,44,45]. Furthermore, women and unemployed people reported poorer experiences compared with men and employed respondents respectively. While it was not possible to identify a clear definition of the relationship between employment status and patient experience, our results are consistent with previous research investigating gender differences in patient experience [46,47]. Therefore, we suggest that additional consideration should be given also to these disadvantaged subgroups from both a practitioner- and policy-oriented perspective. These indications are even more relevant considering that some of the identified subgroups (i.e. women and people with comorbidities) attributed higher scores of importance to integrated care.

Concerning the engagement profiles, while assigning great importance to integrated care, participants with lower engagement described significantly worse experiences. This result is in line with previous studies that explored the association between the level of patient engagement and patient-assessed quality of care, thus confirming the relationship between these two variables [48,49,50]. Patients’ intrinsic ability to be active players in their care journey, in terms of being informed, confident, and proactive, may shape how they experience and evaluate the quality of healthcare delivery. Interestingly, participants with higher engagement (Eudaimonic Project position of engagement) were not the ones reporting the best experiences when considering the delivery of health services. One reason for this result could be that patients who moved to higher engagement positions may actually be improving their interactions with care providers and thus enhancing the care they receive through better utilisation of appropriate planned care, such as self-management support. This could lead patients to be more “critical” and have higher expectations towards the received care.

When accounting for the geographical provenience of participants, no significant differences were observed in the experiences and priorities reported. This result was somehow unexpected as we thought that the historical regional differences in the provision and quality of care across Italy would also be reflected in our data. Furthermore, the geographic variable has been shown to represent a key influencing factor also in other Italian integrated care settings [51,52]. The observed conformity could be explained by the fact that, to the best of our knowledge, none of the regional health services of our country has undertaken relevant integrated care initiatives for people with RMDs yet. Some projects may have been activated at a local level, but their impact could have hardly been captured in our study, which involved a national sample. In this sense, further research to investigate the features and outcomes of possible different local initiatives should be performed. Nevertheless, this (low) common starting point could be an opportunity to implement the directives on integrated care of the National Chronicity Plan jointly across the country, without leaving anyone behind.

Finally, another expected impact of this study is to stimulate the assessment of the added value of people-centred integrated healthcare models according to criteria that really matter to patients and citizens, and employing, besides patient experience, other patient-reported measures such as patient outcomes or patient preference information. From our perspective, this could be an effective way to realise the principles of evidence-based patient advocacy [53], which involves the use of patient-sensitive research to influence healthcare policies.

Our study was subject to some limitations. First, due to the convenience sampling approach adopted, the study sample cannot be considered either random or fully representative of the entire population of Italian people living with RMDs. For example, since part of the participants belonged to a patient organisation (i.e. Apmarr APS), they might have had higher levels of health engagement compared to the general population.

Another limitation is that, since we could not find any patient experience survey suitable for our research, we had to develop our own. While the survey was reviewed for content and face validity and received additional validation through an explorative factor analysis, it did not undergo a complete and rigorous validation process. In this sense, a pilot test of the survey, which could have further modified and improved the questionnaire before its submission to the study participants, was not carried out. Future research could address this issue by administrating the survey to a new population and performing additional psychometric testing to ensure its robustness. This could corroborate both survey validity (e.g. implementing also a confirmatory factor analysis) and reliability (e.g. using a test-retest measure).

Finally, due to privacy issues, we could not know the whole number of people who were contacted to join in our research. This means that it was not possible to collect data on who decided not to participate and thus compare the characteristics of this population with those of our sample.

## Conclusions

Italians with RMDs attributed high importance to integrated care. Studying their experiences of integrated care, a person-centred approach of health professionals and providers was observed. However, when considering the delivery of healthcare services, several shortcomings and difficulties were reported. Comprehensive quality improvement interventions are needed in different areas of service delivery. Particular attention should be paid to frail and/or disadvantaged subgroups and those with lower levels of engagement in their healthcare management. More research to provide additional evidence on the patient experience of integrated care in the context of RMDs is required.

## Additional File

The additional file for this article can be found as follows:

10.5334/ijic.6616.s1 Appendix. Survey questions.
