# Supplementary material for: Patient Experience of Integrated Care: Findings from a Cross-Sectional Study Involving People with Rheumatic and Musculoskeletal Diseases
Source: Int J Integr Care. 2023 May 10;23(2):14. doi: 10.5334/ijic.6616 (PMC10178639; doi:10.5334/ijic.6616)

APPENDIX: Survey questions

Section one: Patient experience of integrated care

Q1. Be considered as a whole by health professionals (providing for your physical, mental, spiritual, and social needs)

How often did it happen to you?

1<br>Never	2<br>Rarely	3<br>Sometimes	4<br>Often	5<br>Always

How important is it for you?

1<br>Not important	2<br>Slightly important	3<br>Moderately important	4<br>Important	5<br>Very important

Q2. Benefit from healthcare services that guarantee access to everyone without discrimination or distinctions of any kind

How often did it happen to you?

1<br>Never	2<br>Rarely	3<br>Sometimes	4<br>Often	5<br>Always

How important is it for you?

1<br>Not important	2<br>Slightly important	3<br>Moderately important	4<br>Important	5<br>Very important

Q3. Be involved by health professionals in the decision-making about your care and treatment programmes

How often did it happen to you?

1<br>Never	2<br>Rarely	3<br>Sometimes	4<br>Often	5<br>Always

How important is it for you?

1<br>Not important	2<br>Slightly important	3<br>Moderately important	4<br>Important	5<br>Very important

Q4. Benefit from healthcare services that guarantee the continuity of health professionals following the patient

How often did it happen to you?

1<br>Never	2<br>Rarely	3<br>Sometimes	4<br>Often	5<br>Always

How important is it for you?

1<br>Not important	2<br>Slightly important	3<br>Moderately important	4<br>Important	5<br>Very important

Q5. Be followed by a general practitioner available to listen to your questions, doubts, and concerns about your health status and/or about the treatments you receive whenever you need it

How often did it happen to you?

1<br>Never	2<br>Rarely	3<br>Sometimes	4<br>Often	5<br>Always

How important is it for you?

1<br>Not important	2<br>Slightly important	3<br>Moderately important	4<br>Important	5<br>Very important

Q6. Be followed by rheumatology specialists (e.g., a rheumatologist) available to listen to your questions, doubts, and concerns about your health status and/or about the treatments you receive whenever you need it

How often did it happen to you?

1<br>Never	2<br>Rarely	3<br>Sometimes	4<br>Often	5<br>Always

How important is it for you?

1<br>Not important	2<br>Slightly important	3<br>Moderately important	4<br>Important	5<br>Very important

Q7. Be contacted by your general practitioner to invite you for a medical check-up or follow-up examination

In the last 12 months, how often did it happen to you?

1<br>Never	2<br>Rarely	3<br>Sometimes	4<br>Often	5<br>Always

How important it is for you?

1<br>Not important	2<br>Slightly important	3<br>Moderately important	4<br>Important	5<br>Very important

Q8. Be contacted by a rheumatology specialist (e.g., a rheumatologist) to invite you for a medical check-up or follow-up examination

In the last 12 months, how often did it happen to you?

1<br>Never	2<br>Rarely	3<br>Sometimes	4<br>Often	5<br>Always

How important it is for you?

1<br>Not important	2<br>Slightly important	3<br>Moderately important	4<br>Important	5<br>Very important

Q9. Have the rheumatology clinics you need near where you live

In the last 12 months, how often did it happen to you?

1<br>Never	2<br>Rarely	3<br>Sometimes	4<br>Often	5<br>Always

How important it is for you?

1<br>Not important	2<br>Slightly important	3<br>Moderately important	4<br>Important	5<br>Very important

Q10. Have the possibility to be examined at home by your general practitioner if you need it

In the last 12 months, how often did it happen to you?

1<br>Never	2<br>Rarely	3<br>Sometimes	4<br>Often	5<br>Always

How important it is for you?

1<br>Not important	2<br>Slightly important	3<br>Moderately important	4<br>Important	5<br>Very important

Q11. Have the possibility to be examined at home by a rheumatology specialist (e.g., a rheumatologist) if you need it

In the last 12 months, how often did it happen to you?

1<br>Never	2<br>Rarely	3<br>Sometimes	4<br>Often	5<br>Always

How important it is for you?

1<br>Not important	2<br>Slightly important	3<br>Moderately important	4<br>Important	5<br>Very important

Q12. Have short waiting times to be examined by your general practitioner

In the last 12 months, how often did it happen to you?

1<br>Never	2<br>Rarely	3<br>Sometimes	4<br>Often	5<br>Always

How important it is for you?

1<br>Not important	2<br>Slightly important	3<br>Moderately important	4<br>Important	5<br>Very important

Q13. Have short waiting times to be examined by a rheumatology specialist (e.g., a rheumatologist)

In the last 12 months, how often did it happen to you?

1<br>Never	2<br>Rarely	3<br>Sometimes	4<br>Often	5<br>Always

How important it is for you?

1<br>Not important	2<br>Slightly important	3<br>Moderately important	4<br>Important	5<br>Very important

Q14. Have the possibility to choose a specific doctor for your rheumatology examination

In the last 12 months, how often did it happen to you?

1<br>Never	2<br>Rarely	3<br>Sometimes	4<br>Often	5<br>Always

How important it is for you?

1<br>Not important	2<br>Slightly important	3<br>Moderately important	4<br>Important	5<br>Very important

Q15. Have the possibility to choose the day and time of a rheumatology examination according to your schedule

In the last 12 months, how often did it happen to you?

1<br>Never	2<br>Rarely	3<br>Sometimes	4<br>Often	5<br>Always

How important it is for you?

1<br>Not important	2<br>Slightly important	3<br>Moderately important	4<br>Important	5<br>Very important

Q16. Have the possibility to book a rheumatology examination online

In the last 12 months, how often did it happen to you?

1<br>Never	2<br>Rarely	3<br>Sometimes	4<br>Often	5<br>Always

How important it is for you?

1<br>Not important	2<br>Slightly important	3<br>Moderately important	4<br>Important	5<br>Very important

Q17. Find all the data, information, and documents you need in a electronic health record

In the last 12 months, how often did it happen to you?

1<br>Never	2<br>Rarely	3<br>Sometimes	4<br>Often	5<br>Always

How important it is for you?

1<br>Not important	2<br>Slightly important	3<br>Moderately important	4<br>Important	5<br>Very important

Q18. Have the possibility to be examined by your general practitioner via video call (e.g., Skype or Zoom)

In the last 12 months, how often did it happen to you?

1<br>Never	2<br>Rarely	3<br>Sometimes	4<br>Often	5<br>Always

How important it is for you?

1<br>Not important	2<br>Slightly important	3<br>Moderately important	4<br>Important	5<br>Very important

Q19. Have the possibility to be examined by a rheumatology specialist (e.g., a rheumatologist) via video call (e.g., Skype or Zoom)

In the last 12 months, how often did it happen to you?

1<br>Never	2<br>Rarely	3<br>Sometimes	4<br>Often	5<br>Always

How important it is for you?

1<br>Not important	2<br>Slightly important	3<br>Moderately important	4<br>Important	5<br>Very important

Section two: Patient Health Engagement Scale (PHE-s®)

Q20. Following, you will find 5 statements that describe how a person might feel when thinking about his/her disease. Each sentence can be completed by indicating one of the 4 states or intermediate points between two states. Please, indicate the state that better describes you by indicating the corresponding position.

Thinking about my health status…							
1	I feel psychologically frozen and blocked		I feel alert		I am aware		I feel positive
	O	O	O	O	O	O	O
2	I feel dazed		I feel worried		I am conscious		I feel peaceful
	O	O	O	O	O	O	O
3	When I think about my illness, I feel overwhelmed by emotions		I feel anxious every time a new symptom arises		I am used to my illness		Despite my illness, my life does not change much. OR I find my life meaningful despite my illness
	O	O	O	O	O	O	O
4	I feel very discouraged due to my illness		I feel anxious when I try to manage my illness		I have adjusted to my illness		I feel optimistic about my future
	O	O	O	O	O	O	O
5	I feel totally oppressed by my illness		I feel upset when a new symptom arises		I have accepted my illness		I have a sense of purpose despite my illness
	O	O	O	O	O	O	O

Section three: Respondent characteristics

21.Gender:

- ☐ Female
- ☐ Male

22.Age:

- ☐ 18-34
- ☐ 35-49
- ☐ 50-64
- ☐ 65 +

23. Region of residence:

- ☐ Northern Italy
- ☐ Central Italy
- ☐ South Italy

24. Marital status:

- ☐ Married
- ☐ Divorced
- ☐ Single

25. Education:

- ☐ University degree
- ☐ High school diploma
- ☐ Middle school diploma

26. Employment:

- ☐ Employed
- ☐ Retired
- ☐ Unemployed

27. How far away do you live from the rheumatology clinics where you are usually examined?

- ☐ More than 50km
- ☐ Between 20 km and 50km
- ☐ Less than 20 km

28. How many years have you suffered from your rheumatic condition(s)?

- ☐ More than 10 years
- ☐ Between 3 and 10 years
- ☐ Less than 3 years

29. Besides your rheumatic condition(s), do you suffer from any other diseases?

- ☐ Yes
- ☐ No

30. Self-reported health

We would like to know how good or bad your health is TODAY.

- This scale is numbered from 0 to 100.
- 100 means the best health you can imagine.
- 0 means the worst health you can imagine.
- Mark an X on the scale to indicate how your health is TODAY.

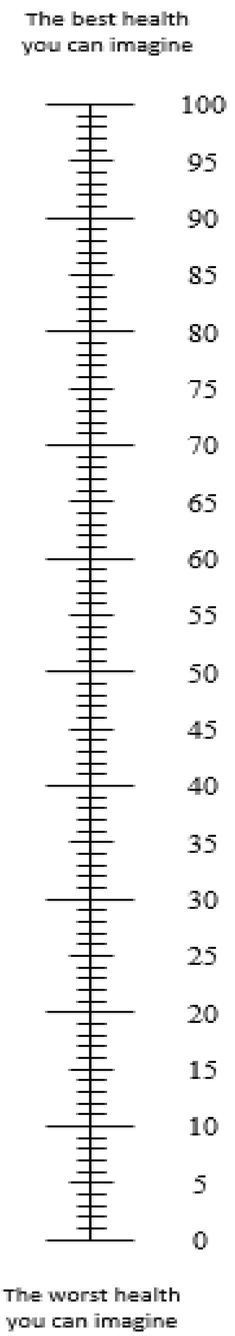

Supplement: Appendix. — Survey questions. [file ijic-23-2-6616-s1.pdf]
